# Exploration of *Streptococcus* core genome to reveal druggable targets and novel therapeutics against *S*. *pneumoniae*

**DOI:** 10.1371/journal.pone.0272945

**Published:** 2022-08-18

**Authors:** Zeshan Mahmud Chowdhury, Arittra Bhattacharjee, Ishtiaque Ahammad, Mohammad Uzzal Hossain, Abdullah All Jaber, Anisur Rahman, Preonath Chondrow Dev, Md. Salimullah, Chaman Ara Keya

**Affiliations:** 1 Bioinformatics Division, National Institute of Biotechnology, Dhaka, Bangladesh; 2 Department of Biochemistry & Microbiology, North South University, Dhaka, Bangladesh; 3 Molecular Biotechnology Division, National Institute of Biotechnology, Dhaka, Bangladesh; University of Malaya Faculty of Medicine, MALAYSIA

## Abstract

*Streptococcus pneumoniae (S*. *pneumoniae)*, the major etiological agent of community-acquired pneumonia (CAP) contributes significantly to the global burden of infectious diseases which is getting resistant day by day. Nearly 30% of the *S*. *pneumoniae* genomes encode hypothetical proteins (HPs), and better understandings of these HPs in virulence and pathogenicity plausibly decipher new treatments. Some of the HPs are present across many *Streptococcus* species, systematic assessment of these unexplored HPs will disclose prospective drug targets. In this study, through a stringent bioinformatics analysis of the core genome and proteome of *S*. *pneumoniae* PCS8235, we identified and analyzed 28 HPs that are common in many *Streptococcus* species and might have a potential role in the virulence or pathogenesis of the bacteria. Functional annotations of the proteins were conducted based on the physicochemical properties, subcellular localization, virulence prediction, protein-protein interactions, and identification of essential genes, to find potentially druggable proteins among 28 HPs. The majority of the HPs are involved in bacterial transcription and translation. Besides, some of them were homologs of enzymes, binding proteins, transporters, and regulators. Protein-protein interactions revealed HP PCS8235_RS05845 made the highest interactions with other HPs and also has TRP structural motif along with virulent and pathogenic properties indicating it has critical cellular functions and might go under unconventional protein secretions. The second highest interacting protein HP PCS8235_RS02595 interacts with the Regulator of chromosomal segregation (RocS) which participates in chromosome segregation and nucleoid protection in *S*. *pneumoniae*. In this interacting network, 54% of protein members have virulent properties and 40% contain pathogenic properties. Among them, most of these proteins circulate in the cytoplasmic area and have hydrophilic properties. Finally, molecular docking and dynamics simulation demonstrated that the antimalarial drug Artenimol can act as a drug repurposing candidate against HP PCS8235_RS 04650 of *S*. *pneumoniae*. Hence, the present study could aid in drugs against *S*. *pneumoniae*.

## Introduction

Community-acquired pneumonia (CAP) is one of the prime causes of death from infectious diseases worldwide [1]. It is an acute lung infection caused by a variety of microorganisms that cause symptoms such as shortness of breath, coughing, heavy sputum, fever, chills, and chest pain [2]. Currently, lower respiratory tract infection (LRTI) is considered to be the fourth-largest cause of death worldwide (https://www.who.int/news-room/fact-sheets/detail/the-top-10-causes-of-death), and CAP is still the leading cause of death among all infectious diseases in the United States of America [1]. Earlier studies have shown that about 900 000 children <5 years died from pneumococcal diseases (https://www.who.int/en/news-room/fact-sheets/detail/pneumonia) and 2.2–50.9% of cases belonged to pediatric CAP [3].

*S*. *pneumoniae* also known as pneumococcus is identified as the most recognizable causative agent of CAP in individuals with a compromised immune system [4]. In 2016, LRTI by *S*. *pneumoniae* engendered 1, 517, 388 deaths globally [5]. Between 1918 and 1919, pneumococcus played a dominant role in the global influenza pandemic [6]. Misuse of pneumonia-related antibiotics during the COVID-19 pandemic has the potential to generate more multi-drug resistant *S*. *pneumoniae* [7]. The fatality of CAP depends on the age and the existence of the comorbidities. The infection is more common among adults aged over 65 years and children under 2 years, or individuals who smoke, have asthma, or have Chronic obstructive pulmonary disease (COPD) [8, 9]. Apart from CAP, *S*. *pneumoniae* also causes bacterial meningitis, bacteremia, sinusitis, otitis media, septic arthritis, aortitis, gingival lesions, phlegmonous gastritis, inguinal adenitis, testicular and tubo-ovarian abscesses, and necrotizing fasciitis [10].

Currently, the therapeutics offered for patients with pneumonia are antibiotic therapies and vaccines. The application of these treatments varies from place to place and also on the severity of the disease. Usually, if the disease is not treated at the right time or is caused by virulent/ resistant strains; it leads to septic shock, empyema, parapneumonic effusion, lung abscess, necrotizing pneumonia, and often death [11–13]. There are two types of vaccines in the global market: the pneumococcal polysaccharide vaccine (23-valent pneumococcal polysaccharide vaccine (PPSV23), and the pneumococcal conjugate vaccine (10-valent PCV10 and 13-valent PCV13) [14, 15]. Owing to its poor immunogenicity in infants, PPSV23 is only recommended for adults and immunocompromised patients while PCV 13 is advised for infants <2 years old. Although PPSV23 has been widely used for over 40 years, there are some limitations. The antibody responses produced by this vaccine are T-cell independent and it was observed that the level of serotype-specific IgG and Opsonophagocytic killing Assay (OPA) lessens over time, hence revaccination is required for protection at an interval of 5 years [16]. Moreover, PPSV23 is less effective in men compared to women [17]. Thus, the efficacy of this vaccine is still unclear. Also, the existing vaccines had a major effect on CAP etiology giving rise to new *S*. *pneumoniae* serotypes. Currently, 98 serotypes with different polysaccharide capsules have been acknowledged [18]. Notably, capsule polysaccharide-based vaccines are limited to a certain number of serotypes.

Over the last few decades, a significant rise in antimicrobial resistance was observed in *S*. *pneumoniae* [19, 20]. This rise is associated with the emergence of multiple serotypes of the bacteria in various parts of the world [21]. Pneumococcus can undergo recombination-mediated genetic plasticity which allows them to acquire antibiotic resistance genes from several closely related species like *Streptococcus mitis* and *Streptococcus oralis* [22]. This in turn helps them evade vaccines and antimicrobial agents and evolve into new vaccine-escape mutants and high antimicrobial-resistant strains [23, 24]. The standard antimicrobial agent approved for CAP is a combination of β-lactam plus a macrolide or fluoroquinolone. β-lactam works by binding to the penicillin-binding proteins (PBP) of the bacteria and inhibits cell wall synthesis. Nevertheless, mutations in the mosaic genes encoding Penicillin-Binding Protein (PBP) results in resistant isolates [25]. Macrolide resistance and resistance to fluoroquinolones originate from the overuse of these broad-spectrum antibiotics. The level of macrolide resistance varies from region to region. North America and the United Kingdom are prone to low macrolide resistance by drug efflux whereas Asian countries are liable to high macrolide resistance as a result of ribosomal methylation [26]. Generally, fluoroquinolone is the only antibiotic used to target the DNA gyrase of *S*. *pneumoniae* directly bringing a halt to its protein synthesis. However, repeated use of this antimicrobial agent resulted in a spontaneous number of mutations in the chromosomal genes that encode this enzyme [27]. As a result, the growing resistance of *S*. *pneumoniae* to commonly used antibiotics and non-vaccine serotypes underlines the urgent need for a new therapeutic target.

Examination of the whole genome of the organism is an important approach in combating the regulation of antibiotic resistance, non-vaccine serotypes, and developments of therapeutics. When varieties of genus *Streptococcus* strains are aligned together, several core genes can be observed. Around 33% of the *S*. *pneumoniae* genome constitutes uncharacterized proteins documented as Hypothetical proteins (HPs) [28]. These proteins are encoded by computationally predicted open reading frames but lack biochemical and chemical evidence. Although they lack functional characterization, they play a crucial role in biochemical and physiological pathways [22]. Bioinformatics tools and algorithms are very efficient to explore proteins [29, 30]. Previous studies have shown some of the *Streptococcus mutans*’ HPs are critical for antibiotic resistance and biofilm formation [22, 31]. Additionally, this infectious agent produces several virulence factors involved in the survival of the pathogen and the progression of the disease [28]. Therefore, in the present study, we aimed to characterize the HPs encoded by the core genome of *S*. *pneumoniae* using a computational approach to uncover novel targets for drug development. Since the HPs are mutual in many *Streptococcus* species they might be interesting targets.

## Methodology

### Retrieval of the genome sequences

The core genome and proteome of *Streptococcus* strains from different Streptococci species were extracted using the Efficient Database framework for comparative Genome Analyses using BLAST score Ratios (EDGAR 3.0) and visualized by BioCircos [32]. EDGAR 3.0 is software designed to perform genome comparisons using a high throughput approach [33]. *S*. *pneumoniae* PCS8235 (NCBI Reference Sequence: NZ_CM001835.1) was taken as the reference strain. HPs were mined manually from the proteome datasets of the strains.

### Functional enrichment and determination of physicochemical properties of the hypothetical proteins

The functions of the HPs were unveiled using the Gene Ontology Functional Enrichment Annotation Tool (GO FEAT) webserver which works through sequence homology search. The proteins were classified based on the conservation of domains, motifs, families, and superfamilies and categorized via the InterPro, UniProt, European Molecular Biology Laboratory (EMBL), Kyoto Encyclopedia of Genes and Genomes (KEGG), and the National Center for Biotechnology Information (NCBI) databases respectively [34]. The physicochemical properties of the annotated HPs were further documented using the ProtParam tool of Expasy (https://web.expasy.org/protparam/). The parameters of the proteins included the molecular weight, theoretical pI point, amino acid composition, extinction coefficient, instability index, aliphatic index, and grand average of hydropathicity (GRAVY) of the protein [35].

### Analysis of subcellular localization and unconventional protein secretion

CELLO (http://cello.life.nctu.edu.tw/) and PSORTb 3.0 (https://www.psort.org/psortb/) were utilized to predict the subcellular localization of the HPs [36, 37]. Understanding subcellular localization is very important to characterize a protein as a target for a drug or vaccine [38]. The OutCyte 1.0 (http://www.outcyte.com/) and SecretomeP 2.0 (http://www.cbs.dtu.dk/services/SecretomeP/) were also used to reveal the unconventional protein secretion as well as the HPs taking non-classical secretory pathways respectively [39]. OutCyte 1.0 is an online bioinformatics tool that mediates two steps to finally generate the proteins without N-terminal signals [40].

### Identification of essential proteins

All the HPs of *S*. *pneumoniae* were queried against the Database of Essential Genes (DEG) database (http://origin.tubic.org/deg/public/index.php) in search of homologous genes within the inquired sequence [41]. Only the proteins encoded by the query sequences which shared similarities with the essential genes in the DEG database and an E-value of <0.0001 and bit-score >100 as cutoff were designated as essential proteins.

### Virulence properties and pathogenicity

VirulentPred (http://bioinfo.icgeb.res.in/virulent/) server was used to evaluate the virulence activity of the HPs. This online tool is an SVM-based method that calculates a virulence potential score for a given protein [42]. Simultaneously, the tool can distinguish virulent and non-virulent proteins. Moreover, the pathogenic proteins were identified from the functionally annotated proteome using the MP3 tool [43]. This software exploits a combined SVM-HMM approach while identifying proteins from both genomic and metagenomic databases with high accuracy, efficiency, and sensitivity.

### Protein-protein interaction network analysis

The protein-protein interaction (PPI) (https://string-db.org/) network between the functionally annotated HPs was visualized using the STRING database [44]. In this study, the available *S*. *pneumoniae* D39 in the STRING database was selected as the reference genome and an interconnected PPI network was constructed. The basis for the network lay in high-throughput lab experiments, gene expression data, and computational data. The network was built with the default confidence parameters.

### Excavation of druggable proteins

The druggability of the annotated proteins was assessed through Drugbank BlastP [45]. Drugbank (https://go.drugbank.com/) is a comprehensive bioinformatics and cheminformatics resource containing relevant information about drugs and their corresponding targets.

### Assessment of Structural proteins

Models for the selected proteins were obtained from Robetta (https://robetta.bakerlab.org/). Robetta analyzes the putative domains of the submitted sequences and generates 3-dimensional structural models [46]. The models were further evaluated using SWISS Structure assessment (https://swissmodel.expasy.org/). SWISS Structure assessment includes Ramachandran Plots and MolProbity scores [47]. Ramachandran Plots visualize energetically favored regions for backbone dihedral angles against the amino acids present in the protein structure while MolProbity examines the quality of protein models for both nucleic acids and proteins at global and local levels [48].

### Molecular docking simulation

The Canonical Simplified molecular-input line-entry system (SMILES) of the interacted drugs were collected from DrugBank BLASTp. These canonical smiles were converted into PDB files via the CACTUS online smile translator (https://cactus.nci.nih.gov/translate/). The druggable proteins were then docked using AutoDock within PyRx software, which is a combination of several tools necessary for Molecular Docking [49]. The protein-ligand interactions were then visualized using Discovery Studio Visualizer (https://discover.3ds.com/).

### Molecular Dynamics (MD) simulation

To evaluate the stability of the complexes under physiological conditions, 100 ns Molecular Dynamics (MD) simulation was carried out using GROningen MAchine for Chemical Simulations (GROMACS version 5.1.1). The GROMOS96 43a1 force-field was applied to the protein-ligand complexes. The physiological condition of the system was defined as (300 K, pH 7.4, 0.9% NaCl). The structures were solvated in a dodecahedral box of the SPC (simple point charge) water model with its edges at a 1nm distance from the protein surface. The overall charge of the system was neutralized through the addition of 2 sodium ions using the genion module. Energy minimization of the neutralized system was carried out using the steepest descent minimization algorithm with a maximum number of minimization steps to perform was set at 50000. The ligand was restrained before carrying out the isothermal-isochoric (NVT) equilibration of the system for 100 ps with a short-range electrostatic cutoff value of 1.2 nm. Isobaric (NPT) equilibration of the system was carried out for 100 ps following the NVT with a short-range van der Waals cutoff fixed at 1.2 nm. Later, a 10 ns molecular dynamic simulation was run using periodic boundary conditions and a time integration step of 2 fs. The energy of the system was saved every 100 ps. For calculating the long-range electrostatic potential, the Particle Mesh Ewald (PME) method was applied. The short-range van der Waals cutoff was kept at 1.2 A modified Berendsen thermostat was used to control simulation temperature while the pressure was kept constant using the Parrinello-Rahman algorithm. The simulation time step was selected as 2.0 fs. The snapshot interval was set to 100 ps for analyzing the trajectory data. Finally, all of the trajectories were concatenated to calculate and plot root mean square deviation (RMSD), root mean square fluctuation (RMSF), the radius of gyration (Rg), and solvent accessible surface area (SASA) data. Root Mean Square Deviation (RMSD) calculation was performed to evaluate when a system attains equilibrium. The “rms” module built into the GROMACS software was utilized to extract RMSD information throughout the simulation. The results were plotted graphically using the ggplot2 package of R (https://ggplot2.tidyverse.org/).

Room Mean Square Fluctuation (RMSF) is used to determine the flexibility of a certain region of the protein. The radius of gyration of our proteins was measured to determine their degree of compactness. A relatively steady value of the radius of gyration means stable folding of a protein. Fluctuation of the radius of gyration implies the unfolding of the protein. The “gyrate” module was used to generate the radius of gyration graphs for our proteins.

Hydrophobic interactions composed of non-polar amino acids are crucial for maintaining the stability of the hydrophobic core of proteins. They do so by covering the non-polar amino acids within the hydrophobic cores and keeping them at a distance from the solvent. Solvent Accessible Surface Area (SASA) is used in MD simulations to predict the hydrophobic core stability of proteins.

## Results

**Fig 1:**
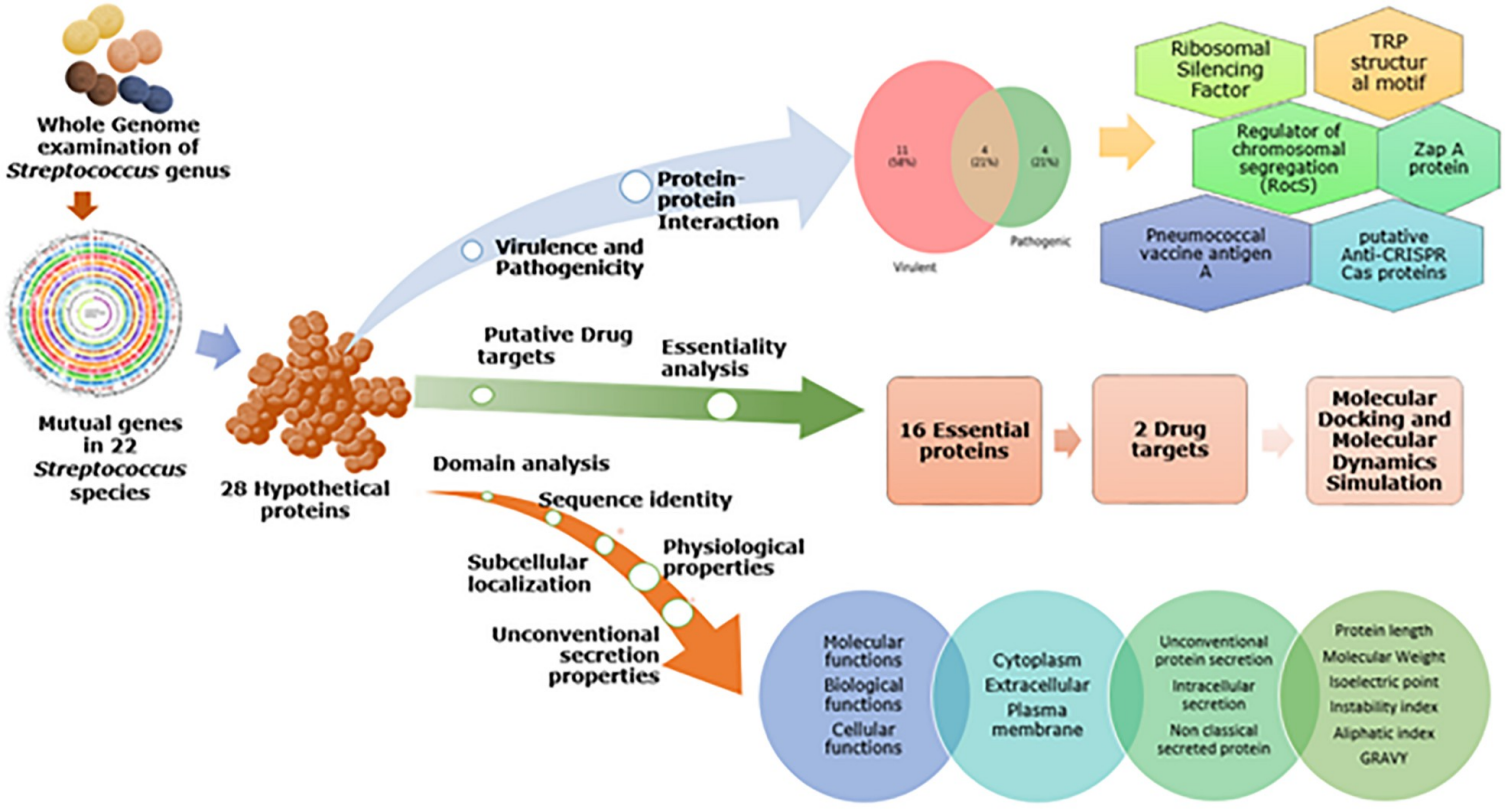
A schematic representation of the workflow involved to identify potential anti-streptococci drug targets.

In the study, the core genome of *S*. *pneumoniae* that encodes hypothetical proteins (HPs) was computationally annotated and analyzed to identify potential drug targets. The workflow has been illustrated in Fig 1.

### Identification of proteins from core and pan genome

**Fig 2:**
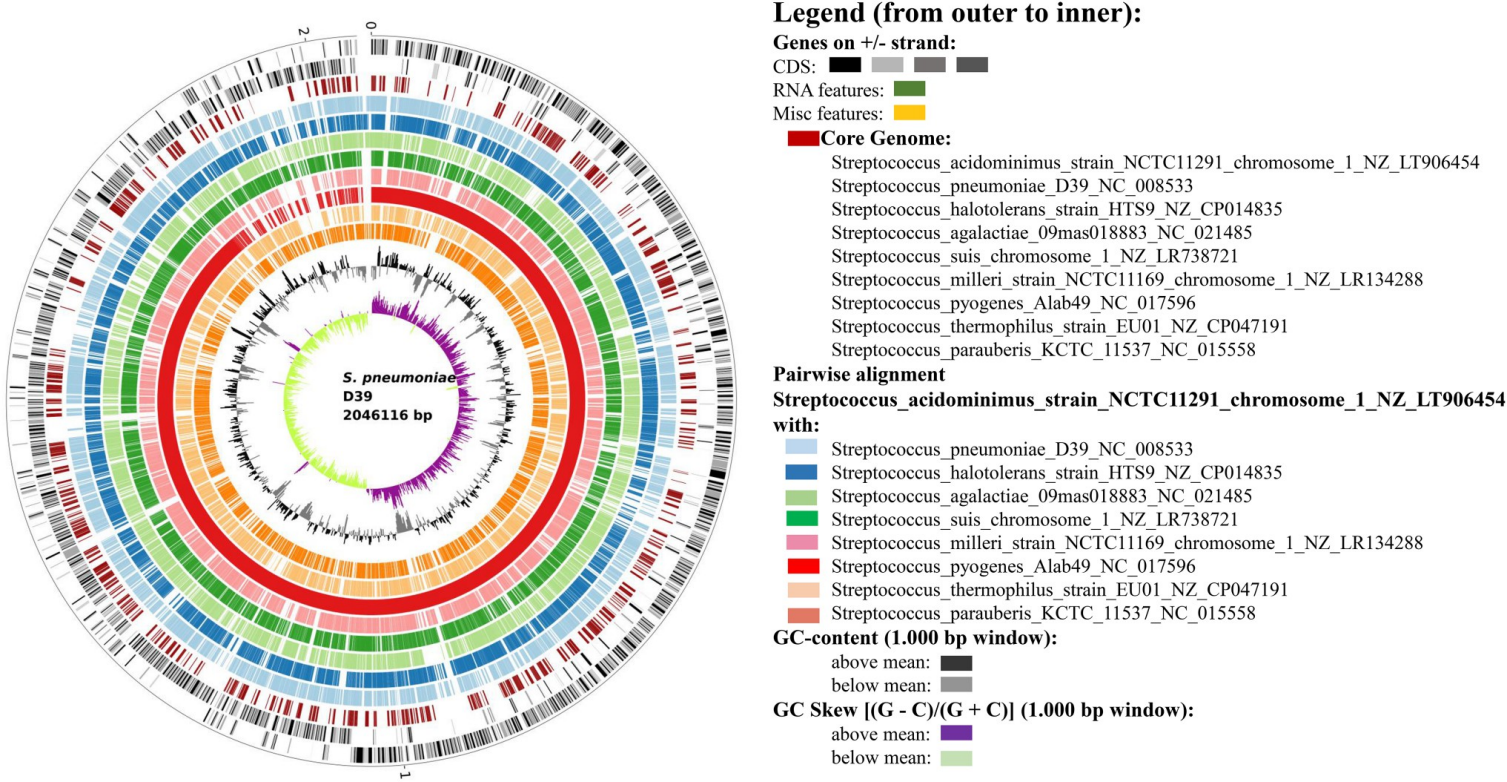
Circular representation of the *S*. *pneumoniae* genome and related species. The brown bar of the circular plot represents the core genes.

Out of 22 different Streptococci species from EDGAR 3.0, 9084 genes from pan genome were identified and several were visualized by the BioCircos tool. Subsequently, 498 genes were extracted from the core genome (S1 Data). The core genome dataset of *S*. *pneumoniae* encoded 28 HPs (Fig 2) (S2 Data).

### Functional analysis of the hypothetical proteins

**Fig 3:**
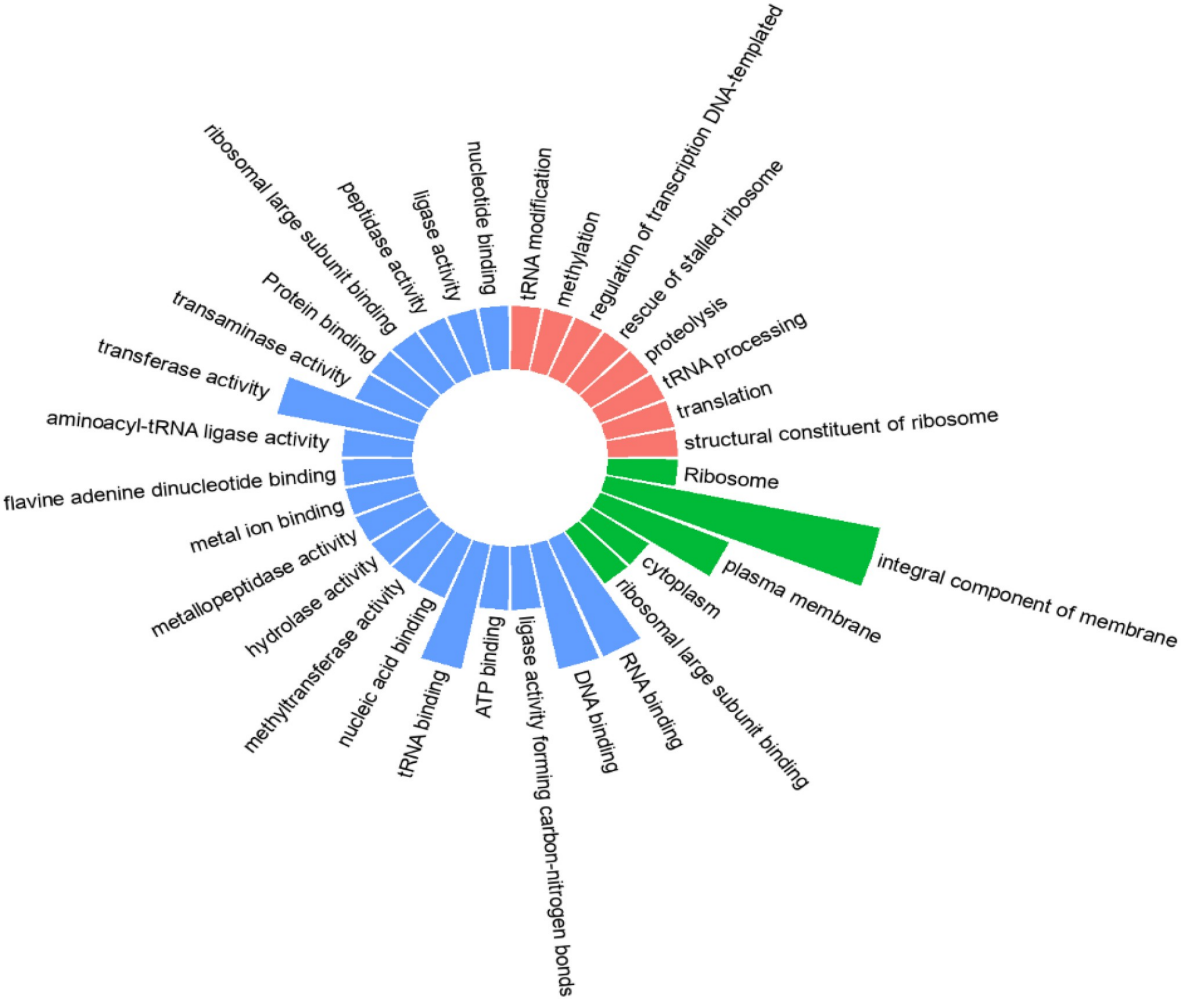
Categories of annotated *S*. *pneumoniae* hypothetical proteins classified according to their biological, molecular, and cellular functions. Proteins involved in molecular functions were relatively higher compared to others.

The gene ontology (GO) analysis of the 28 HPs revealed that 21 annotated proteins had been involved in biological processes (BPs), molecular functions (MFs) and cellular components (CCs) and the remaining belonged to uncharacterized domains/ protein families (S3 Data). The HPs corresponded to 20 GO terms in total while some of the HPs possessed more than one GO term (Fig 3 and Table 1). HPs were homologs of enzymes, binding proteins, transporters, and regulators (Fig 3). A high proportion of HPs exhibited MFs such as aminoacyl-tRNA ligase activity, RNA binding, DNA binding, hydrolase activity, transferase activity, tRNA binding, methylation, nucleic acid binding, ATP binding, metal ions binding, Flavin adenine dinucleotide binding, tRNA modification, and metallopeptidase activity, indicating they may take part in translation and transcription of the bacteria. The study also predicted 6 proteins as members of the integral membrane protein family.

**Table 1:** Annotated products for HPs in *S*. *Pneumoniae* as determined by GO FEAT platform.

Seq no.	Locus Tag	Product	Gene ontology	BlastP
**1**.	PCS8235_RS00650	Uncharacterized protein		MULTISPECIES: cell division protein ZapA [*Streptococcus*]
**2**.	PCS8235_RS01335	W1WQE4_9ZZZZ YlxR domain-containing protein OS = human gut metagenome OX = 408170 GN = Q604_UNBC18436G0004 PE = 4 SV = 1		Cytosolic protein YlxR [*S*. *pneumoniae*]
**3**.	PCS8235_RS01340	Ribosomal protein HS6-type	GO:0003723; GO:0005840;	L7A family ribosomal protein [*S*. *pneumoniae*]
**4**.	PCS8235_RS01935	Uncharacterized protein		DUF910 family protein [*S*. *mitis*]
**5**.	PCS8235_RS02405	Q8DQG2_STRR6 PC4 domain-containing protein OS = *S*. *pneumoniae* (strain ATCC BAA-255 / R6) OX = 171101 GN = spr0690 PE = 1 SV = 1	GO:0003677; GO:0006355	YdbC family protein [*S*. *pneumoniae*]
**6**.	PCS8235_RS02560	Uncharacterized protein		DUF1827 family protein [*S*. *pneumoniae*]
**7**.	PCS8235_RS02595	Uncharacterized protein		Protein of uncharacterised function (DUF1797) [*S*. *pneumoniae*]
**8**.	PCS8235_RS02800	Uncharacterized protein		MULTISPECIES: DUF2969 domain-containing protein [*Streptococcus*]
**9**.	PCS8235_RS02820	GAF domain-containing protein		Structure of fRMsr [*S*. *pneumoniae* R6]
**10**.	PCS8235_RS03050	Membrane protein puative	GO:0005886; GO:0016021	permease [*S*. *pneumoniae*]
**11**.	PCS8235_RS03260	M5KND5_STREE UPF0346 protein PCS8203_00654 OS = *S*. *pneumoniae* PCS8203 OX = 1159082 GN = PCS8203_00654 PE = 3 SV = 1		YozE family protein [*S*. *pneumoniae*]
**12**.	PCS8235_RS03345	M5KNF4_STREE Rqc2 homolog RqcH OS = *S*. *pneumoniae* PCS8203 OX = 1159082 GN = rqcH PE = 3 SV = 1	GO:0000049; GO:0043023; GO:0072344	Rqc2 family fibronectin-binding protein PavA [*S*. *pneumoniae*]
**13**.	PCS8235_RS03470	A0A3R9LSP5_STROR DUF536 domain-containing protein OS = *Streptococcus oralis* OX = 1303 GN = D8800_01470 PE = 4 SV = 1		MULTISPECIES: chromosome segregation protein RocS [*Streptococcus*]
**14**.	PCS8235_RS03660	Pneumococcal vaccine antigen A		pneumococcal vaccine antigen A [*S*. *pneumoniae*]
**15**.	PCS8235_RS03915	DNA repair protein RadC	GO:0046872; GO:0008237	MULTISPECIES: DNA repair protein RadC [*Streptococcus*]
**16**.	PCS8235_RS04650	Haloacid dehalogenase	GO:0016787	Cof-type HAD-IIB family hydrolase [*S*. *pneumoniae*]
**17**.	PCS8235_RS04815	Putative ACR		DUF177 domain-containing protein [*S*. *pneumoniae*]
**18**.	PCS8235_RS05215	M5KML3_STREE UPF0342 protein PCS8203_00480 OS = *S*. *pneumoniae* PCS8203 OX = 1159082 GN = PCS8203_00480 PE = 3 SV = 1		YlbF/YmcA family competence regulator [*S*. *pneumoniae*]
**19**.	PCS8235_RS05845	Tetratricopeptide repeat protein		tetratricopeptide repeat protein [*S*. *pneumoniae*]
**20**.	PCS8235_RS06025	Methyltransferase	GO:0008168; GO:0003676; GO:0032259	tRNA1(Val) (adenine(37)-N6)-methyltransferase [*S*. *pneumoniae*]
**21**.	PCS8235_RS06945	M5MZN5_STREE tRNA(Met) cytidine acetate ligase OS = *S*. *pneumoniae* PNI0446 OX = 1159102 GN = tmcAL PE = 3 SV = 1	GO:0000049; GO:0005524; GO:0005737; GO:0006400; GO:0016879	nucleotidyltransferase [*S*. *pneumoniae*]
**22**.	PCS8235_RS07090	Protein of uncharacterized function (DUF3013)		MULTISPECIES: DUF3013 family protein [*Streptococcus*]
**23**.	PCS8235_RS07595	A0A3R9LRS7_STROR UPF0154 protein D8800_03695 OS = *S*. *oralis* OX = 1303 GN = D8800_03695 PE = 3 SV = 1	GO:0005886; GO:0016021	YneF family protein *[S*. *pneumoniae*]
**24**.	PCS8235_RS08090	A0A1E9G3W9_9STRE UPF0297 protein HMPREF2766_08835 OS = *Streptococcus sp*. HMSC076C08 OX = 1739270 GN = HMPREF2766_08835 PE = 3 SV = 1		MULTISPECIES: IreB family regulatory phosphoprotein [*Streptococcus*]
**25**.	PCS8235_RS08125	Uncharacterized protein	GO:0016021	MULTISPECIES: DUF1129 domain-containing protein [*Streptococcus*]
**26**.	PCS8235_RS08375	M5K7W4_STREE UPF0356 protein PCS8203_00243 OS = *S*. *pneumoniae* PCS8203 OX = 1159082 GN = PCS8203_00243 PE = 3 SV = 1		MULTISPECIES: DNA-dependent RNA polymerase auxiliary subunit epsilon family protein [*Streptococcus*]
**27**.	PCS8235_RS10555	J1NSH0_STREE UPF0374 protein AMCSP13_002284 OS = *S*. *pneumoniae* 2070335 OX = 914141 GN = AMCSP13_002284 PE = 3 SV = 1		UPF0374 protein SSU05 [*S*. *pneumoniae*]
**28**.	PCS8235_RS10805	Uncharacterized protein	GO:0016021 GO:0050660	HlyC/CorC family transporter [*S*. *pneumoniae*]

### Physicochemical characterization

The physicochemical properties of the 28 HPs are provided in Table 2. The length of the analyzed sequence ranged from 71 to 560 AA with a molecular weight (MW) ranging from 8462.34 and 64392.6 Da. The isoelectric point (pI) of the proteins ranged from 3.92 to 10.07 with an average value of 6.20. The isoelectric point determines the protein load. At this pH, the protein does not have any charge and therefore does not move in the electric field when a direct current is passed through it [50]. MW and pI parameters are important for purification and crystallization during *in vivo* experimentation. The extinction coefficient measures the amount of light that a protein absorbs at a certain wavelength. *In silico* identification of the extinction coefficient is necessary to evaluate the study of protein-protein interaction in solution [51]. The extinction coefficient of the provided hypothetical proteins ranged from 1615 to 57190 at 280 nm. Here, HPs possessed high extinction coefficient values that indicate the proteins constitute a high concentration of cysteine, tyrosine, and tryptophan. The instability index determines the stability of the protein in a test tube. An instability index value of less than 40 predicts the protein to be stable and over 40 adopts the protein to be unstable [52]. With variable ranges of instability index, more than 50% of the proteins were noticed to be stable with a mean value of 39.21 in this study. The aliphatic index estimates the thermostability of the protein depending on the area occupied by the aliphatic chain. Proteins with high aliphatic index show stability in high temperatures, whereas proteins with low aliphatic value are less flexible and thermally unstable [53]. Here, the aliphatic index of the proteins ranged from 32.66 to 1135.05 with a mean value of 92.16. Most of the proteins were observed with an aliphatic index value above 40 which means most of them are thermally stable. The GRAVY of the protein indicates the interaction of proteins with water. It is calculated by adding all the hydropathy values of the amino acids followed by dividing the number of residues in the sequence. The outcome for GRAVY predicts the presence of the protein-water interactions between all the proteins except two. Therefore, the majority of them are water-soluble.

**Table 2:** Physicochemical properties of HPs obtained using ProtParam tool.

Locus tag	Protein length	Molecular weight	Isoelectric point	Negatively charged residues	Positively charged residues	extinction coefficient	instability index	Aliphatic index	GRAVY
RS00650	100	11568.13	4.82	20	13	1615	63.16	85.9	-0.559
RS01335	97	11240.04	9.62	16	22	4470	32.66	32.66	-0.663
RS01340	99	10867.78	9.87	9	16	2980	26.31	110.3	-0.035
RS01935	73	8838.32	5.8	14	13	8940	56.25	100.14	-0.44
RS02405	79	9136.44	6.73	13	13	11000	35.8	58.1	-0.765
RS02560	99	11257.94	6.83	12	12	4470	36.29	106.36	-0.255
RS02595	76	8837.87	4.37	17	7	5960	29.95	96.18	-0.333
RS02800	76	8463.82	7.88	12	13	4470	25.25	102.63	-0.283
RS02820	165	18421.04	4.41	25	13	14565	38.13	95.7	0.045
RS03050	301	33319.99	8.58	15	18	24660	33.83	135.05	1.045
RS03260	71	8462.34	4.68	14	7	13980	34.76	56.48	-0.686
RS03345	560	64392.58	8.43	74	77	29465	46.28	91.93	-0.535
RS03470	163	18970.57	4.88	36	28	2980	53.18	89.69	-0.852
RS03660	204	22915.15	9.16	16	21	27850	21.54	87.01	-0.199
RS03915	226	25535.53	6.64	26	24	7450	49.12	111.77	-0.165
RS04650	264	29481.57	4.64	42	25	21890	18.59	94.51	-0.102
RS04815	180	20398.72	3.93	39	12	14440	56.59	99.61	-0.353
RS05215	112	12471.19	4.83	16	12	5960	52.41	86.43	-0.271
RS05845	403	46780.9	4.2	87	28	57190	43.96	95.29	-0.434
RS06025	249	28367.53	5.75	35	30	12170	43.62	96.71	-0.326
RS06945	365	41269.4	7.12	43	43	39880	25.92	94.05	-0.243
RS07090	156	18064.09	3.92	38	10	22920	38.15	81.86	-0.222
RS07595	82	9085.76	10.07	6	13	2980	29.12	115.49	-0.084
RS08090	88	10227.41	4.9	15	12	11920	21.41	82.95	-0.749
RS08125	227	25799	6.42	21	20	32890	43.44	98.9	0.146
RS08375	77	9258.34	4.58	18	11	8940	66.6	83.64	-0.779
RS10555	177	21338.22	6.31	29	27	49850	41.1	80.9	-0.692
RS10805	443	49621.74	4.36	70	35	34380	34.35	110.45	0.071
Average	186.14	21228.26	6.20464	27.78	20.5357	17152.3	39.2060	92.167	-0.3113

### Prediction of essential proteins

Identification of essential genes contributes a principal role in the development of new drugs or vaccines that inhibit the dissemination of resistant strains [54]. Through a similarity search against the essential proteins of bacteria present in the DEG database, 16 HPs were considered as essential proteins with an E-value<0.0001 and bit score>100 as shown in Table 3.

**Table 3:** Essential proteins as identified by the DEG database. More than half of the proteins were found to be essential.

Query ID	Subject Id	E-value	Bit score
**PCS8235_RS00650**	DEG10100355	0.37	28.5
**PCS8235_RS01335**	DEG10070031	1.44E-66	194
**PCS8235_RS01340**	DEG10580251	1.75E-52	159
**PCS8235_RS01935**	DEG10350401	0.82	26.6
**PCS8235_RS02405**	DEG10580070	1.45E-41	129
**PCS8235_RS02560**	DEG10570129	3.2	25.8
**PCS8235_RS02595**	DEG10050604	1.1	26.6
**PCS8235_RS02800**	DEG10580106	8.13E-31	102
**PCS8235_RS02820**	DEG10070045	4.36E-121	337
**PCS8235_RS03050**	DEG10470184	5.09E-80	244
**PCS8235_RS03260**	DEG10580325	6.62E-35	112
**PCS8235_RS03345**	DEG10500070	1.11E-08	57
**PCS8235_RS03470**	DEG10540130	4.16E-78	229
**PCS8235_RS03660**	DEG10480049	0.5	30.4
**PCS8235_RS03915**	DEG10070165	6.91E-166	456
**PCS8235_RS04650**	DEG10580145	6.13E-62	195
**PCS8235_RS04815**	DEG10070175	9.05E-129	358
**PCS8235_RS05215**	DEG10080304	1.1	27.3
**PCS8235_RS05845**	DEG10290281	0.15	33.1
**PCS8235_RS06025**	DEG10500005	4.06E-19	82.8
**PCS8235_RS06945**	DEG10230304	0.37	32
**PCS8235_RS07090**	DEG10320245	2.9	26.6
**PCS8235_RS07595**	DEG10070224	7.65E-55	164
**PCS8235_RS08090**	DEG10070008	9.91E-61	179
**PCS8235_RS08125**	DEG10210140	0.29	31.6
**PCS8235_RS08375**	DEG10070005	4.49E-50	151
**PCS8235_RS10555**	DEG10620252	1.79E-77	228
**PCS8235_RS10805**	DEG10390037	7.16E-50	174

### Virulence factors and pathogenicity

**Fig 4:**
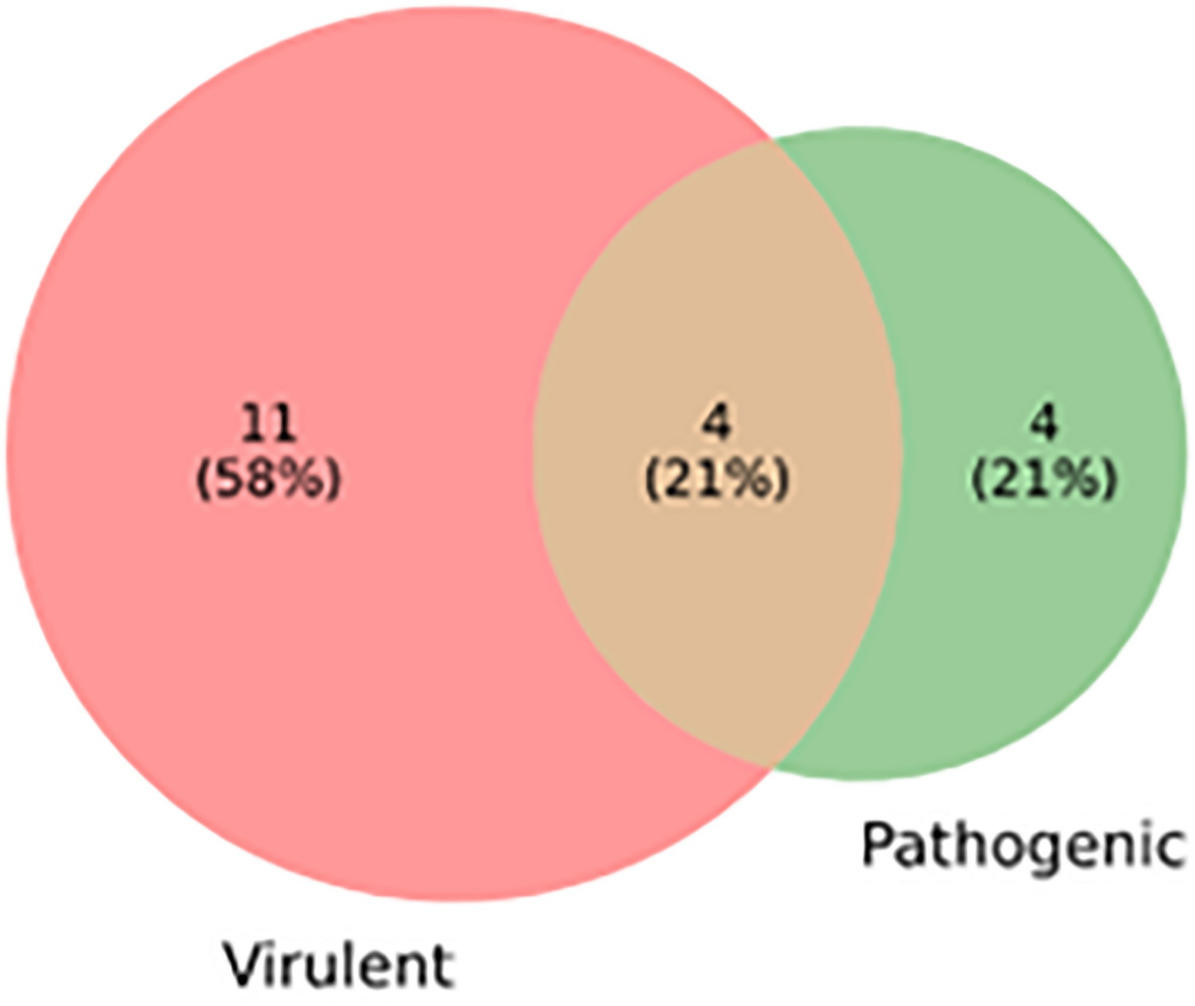
Venn diagram representing the number of hypothetical proteins with virulence factor pathogenicity.

Virulence factors are the basis of bacterial infections. The virulent and pathogenic proteins help microbes invade the host and manipulate the host immune system for its survival [55]. The comprehensive predictive data for virulence is provided. The VirulentPred server identified 54% of the HPs as having virulent properties while 46% owned non virulent properties. Moreover, the MP3 webserver recognized 8 proteins out of 28 as pathogenic. Among these 8 proteins, 4 portrayed both virulence and pathogenic properties (Fig 4) (S4 Data).

### Analysis of the components of the PPI network

**Fig 5:**
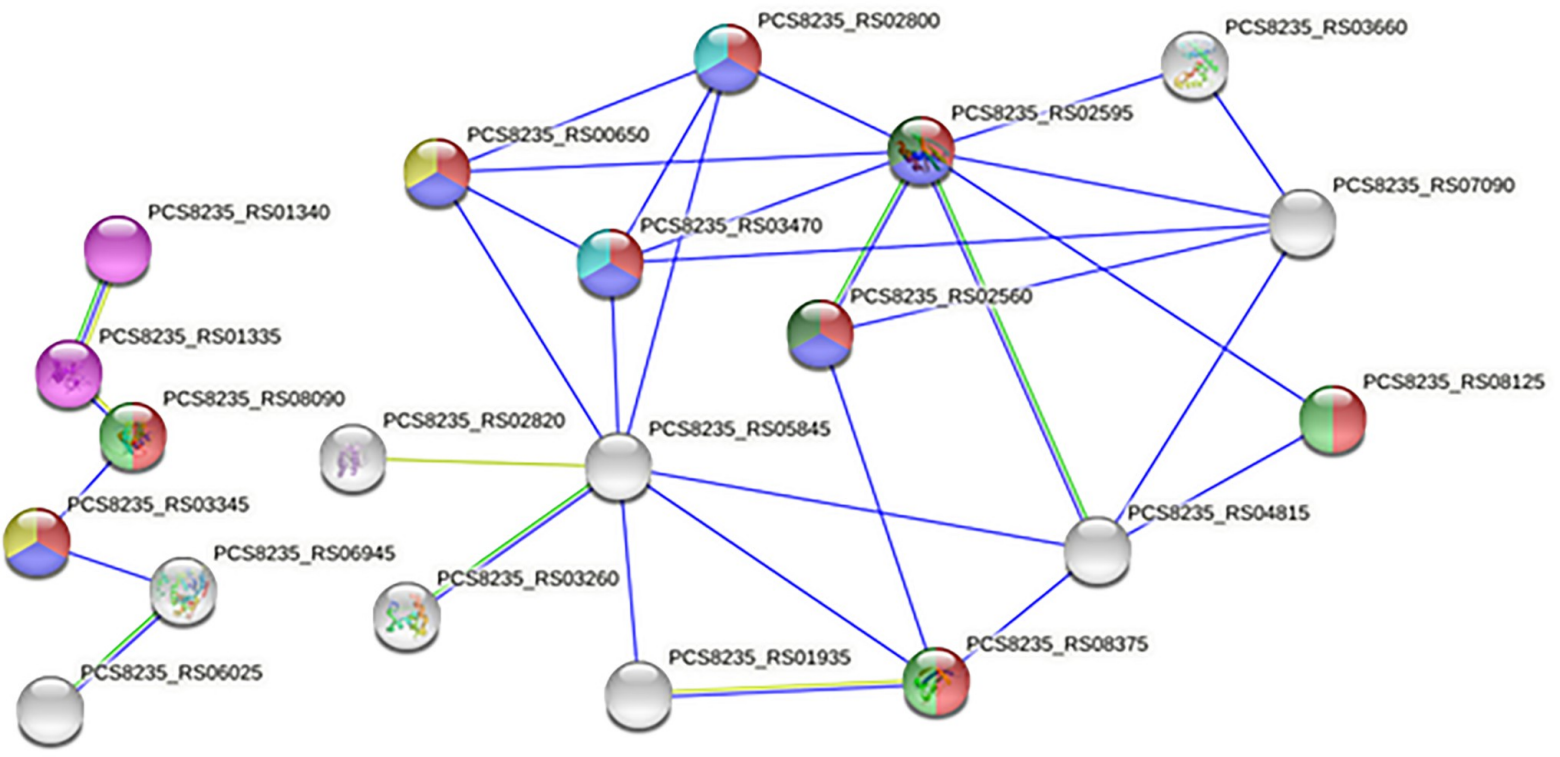
String results of PPI revealed several HPs that interact with pneumococcal vaccine antigen A and Ribosomal silencing factor; PCS8235_RS05845 and PCS8235_RS02595 have the highest nodes.

Protein-Protein interactions are crucial for all cellular functions. The association between the hypothetical proteins of *S*. *pneumoniae* and other proteins is visualized in Fig 5. Among 28 proteins, 20 proteins were involved within the network with a default confidence value. The network contained 28 nodes (proteins) and 32 edges (interactions). However, two of the proteins (PCS8235_RS05845 and PCS8235_RS02595) were observed to have the highest number of interactions with other proteins. The network also revealed some proteins that interact with Pneumococcal vaccine antigen A, Ribosomal Silencing Factor, Cof family domain, CBS domain, and cell division protein Zap A, putative Anti-CRISPR, Regulator of chromosomal segregation (RocS) proteins, and so on. Further analysis of the functional enrichments in the network exhibited proteins that work as Ras family proteins, NusA-like KH domain, Putative fluoride ion transporter CrcB and P-loop containing nucleoside triphosphate hydrolase (S5 Data).

### Examination of the subcellular localization and unconventional protein secretion

During drug discovery, knowledge of subcellular localization can be leveraged to significantly improve the identification of druggable targets [56]. Proteins are categorized as drugs or vaccine targets based on subcellular location. Considering the results of the CELLO and PSORTb 3.0 tools, the majority of the proteins were characterized as cytoplasmic and 6 as membrane-bound with a sole exception being located in the extracellular matrix (Table 4). This implies that most of the proteins prefer the cytoplasmic region for their activity. Furthermore, OutCyte 1.0 data predicted that 64% of all the HPs undergo potential unconventional protein secretion (UPS) and the rest possessed signal peptide, the transmembrane domain, and intracellular secretion. Additionally, SecretomeP 2.0 identified 20 non classical secreted proteins. These proteins lacked a signal peptide and had a threshold SecP value above 0.5. All the predicted results for CELLO, PSORTb 3.0, OutCyte 1.0, and SecretomeP 2.0 are presented in Table 4.

**Table 4:** Prediction of subcellular localization and unconventional protein secretion in HPs by CELLO, PSORTb, OutCyte 1.0 and SecretomeP 2.0.

Seq ID	CELLO	PSORTb 3.0	Outcyte 1.0 Class	SecretomeP 2.0 score
RS00650	cytoplasmic	Cytoplasmic	UPS	0.075661
RS01335	cytoplasmic	Cytoplasmic	Intracellular	0.444115
RS01340	cytoplasmic	Cytoplasmic Membrane	UPS	0.074983
RS01935	cytoplasmic	Cytoplasmic	UPS	0.047038
RS02405	cytoplasmic	Cytoplasmic	UPS	0.702673
RS02560	cytoplasmic + extracellular	Cytoplasmic	UPS	0.353897
RS02595	cytoplasmic	Cytoplasmic	Intracellular	0.049599
RS02800	cytoplasmic	Cytoplasmic	UPS	0.762960
RS02820	cytoplasmic	unknown	UPS	0.071492
RS03050	membrane	Cytoplasmic Membrane	Transmembrane	0.970836
RS03260	cytoplasmic	unknown	UPS	0.215002
RS03345	cytoplasmic	cytoplasmic	Intracellular	0.200677
RS03470	cytoplasmic	cytoplasmic	UPS	0.089014
RS03660	extracellular	Cytoplasmic Membrane	Signal peptide	0.495995
RS03915	membrane	cytoplasmic	UPS	0.122882
RS04650	cytoplasmic	cytoplasmic	UPS	0.05868
RS04815	cytoplasmic	cytoplasmic	UPS	0.132968
RS05215	cytoplasmic	cytoplasmic	UPS	0.269975
RS05845	cytoplasmic	cytoplasmic	Intracellular	0.153385
RS06025	cytoplasmic	cytoplasmic	UPS	0.10396
RS06945	cytoplasmic	cytoplasmic	Intracellular	0.098969
RS07090	cytoplasmic	cytoplasmic	UPS	0.104500
RS07595	membrane	Cytoplasmic Membrane	Signal peptide	0.933010
RS08090	cytoplasmic	unknown	UPS	0.829525
RS08125	membrane	Cytoplasmic Membrane	UPS	0.961769
RS08375	cytoplasmic	cytoplasmic	Intracellular	0.090707
RS10555	cytoplasmic	cytoplasmic	UPS	0.235292
RS10805	membrane	Cytoplasmic Membrane	Signal peptide	0.720349

*UPS- unconventional protein secretion

### Druggability of the proteins

The druggability prediction of HPs classified three proteins with homologous genes (PCS8235_RS02820, PCS8235_RS04650, and PCS8235_RS10805) available in the Drugbank database (Table 5). DrugBank contains arrays of gene families that have been successfully targeted by drugs with required affinity. One of the HPs (PCS8235_RS04650) had two homologous compounds. Three of the predictive druggable proteins were filtered into two (PCS8235_RS02820, PCS8235_RS04650) based on a cut-off E-value of <0.0001 and bit-score >100.

**Table 5:** Homologs of selected HPs found interacting with existing drugs in Drug Bank.

Sequence ID	Homologous compound	E value	Bit score:	Interactions with drug
PCS8235_RS02820	Free methionine-R-sulfoxide reductase	4.90E-37	124.02	2-(N-morpholino) ethanesulfonic acid
PCS8235_RS04650	Haloacid dehalogenase-like hydrolase	1.21E-11	62.003	Artenimol
PCS8235_RS04650	Sugar phosphatase YbiV	5.74E-31	114.775	Aspartate beryllium trifluoride
PCS8235_RS10805	Metal transporter CNNM2	4.16E-13	70.0922	Magnesium carbonate

### Protein model building

Robetta predicted 5 models for each druggable protein. Robetta modeling program was implemented to predict the best model among all. The Ramachandran plot percentage and the Molprobity score of the proteins are tabulated below (S6 Data). MolProbity score should be as low as possible and Ramachandran plot >98% is favored for an ideal protein structure. Model 2 for both of the proteins was selected using these criteria. Model 2 of both of the proteins (PCS8235_RS02820 and PCS8235_RS04650) were selected based on Ramachandran plot percentages of 98.77% and 99.24% with a low morbidity score of 2.64 and 2.69 respectively.

### Elucidation of molecular docking

**Fig 6:**
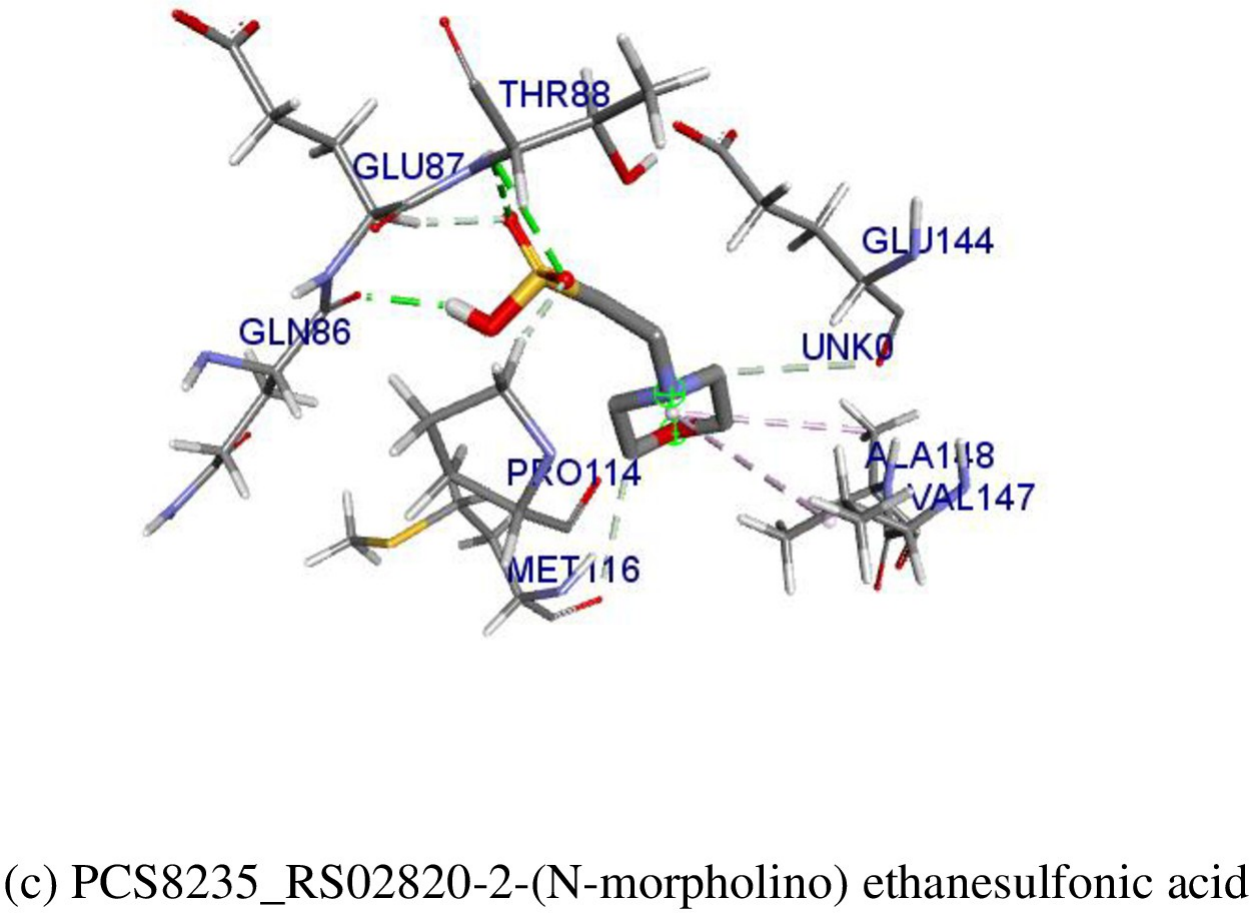
Interaction between the best drug for the HPs in *S*. *pneumoniae* and the active site amino acid residues of each of the HPs (a) PCS8235_RS04650- Artenimol (b) PCS8235_RS04650- Aspartate beryllium trifluoride (c) PCS8235_RS02820-2-(N-morpholino) ethanesulfonic acid.

Blind docking was performed to evaluate the binding energy between each ligand and receptors. Two of the expected potential HPs, PCS8235_RS02820 and PCS8235_RS04650 were docked against the interacted drugs from DrugBank (Fig 6). The binding affinity between druggable targets and commercially available drugs was satisfactory. Protein PCS8235_RS02820 docked against 2-(N-morpholino) ethanesulfonic acid with docking energy of -4.4 kcal/mol. Protein PCS8235_RS 04650 bonded with Artenimol and Aspartate beryllium trifluoride with the binding affinity of -9.5 kcal/mol and -4.7 kcal/mol respectively.

### Examination of molecular dynamics simulation

**Fig 7:**
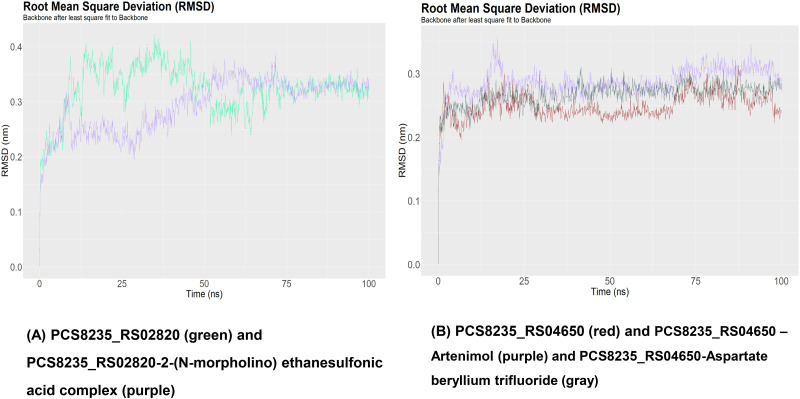
RMSD analysis of (A) PCS8235_RS02820 (green) and PCS8235_RS02820-2-(N-morpholino) ethanesulfonic acid complex (purple) (B) PCS8235_RS04650 (red) and PCS8235_RS04650 –Artenimol (purple) and PCS8235_RS04650-Aspartate beryllium trifluoride (gray) at 100ns.

**Fig 8:**
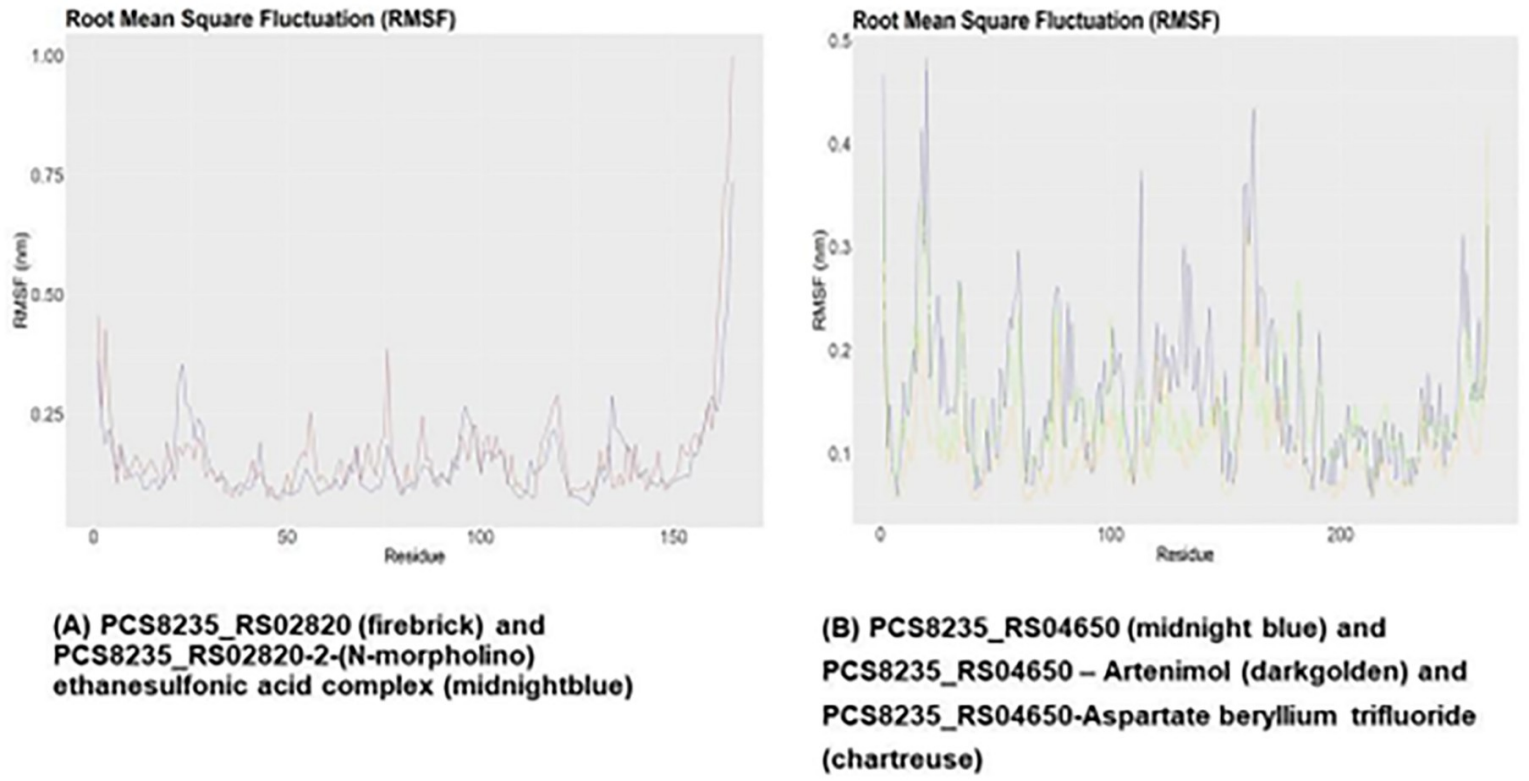
RMSF analysis of (A) PCS8235_RS02820 (brown) and PCS8235_RS02820-2-(N-morpholino) ethanesulfonic acid complex (blue) (B) PCS8235_RS04650 (blue) and PCS8235_RS04650 –Artenimol (golden) and PCS8235_RS04650-Aspartate beryllium trifluoride (green) at 100 ns.

**Fig 9:**
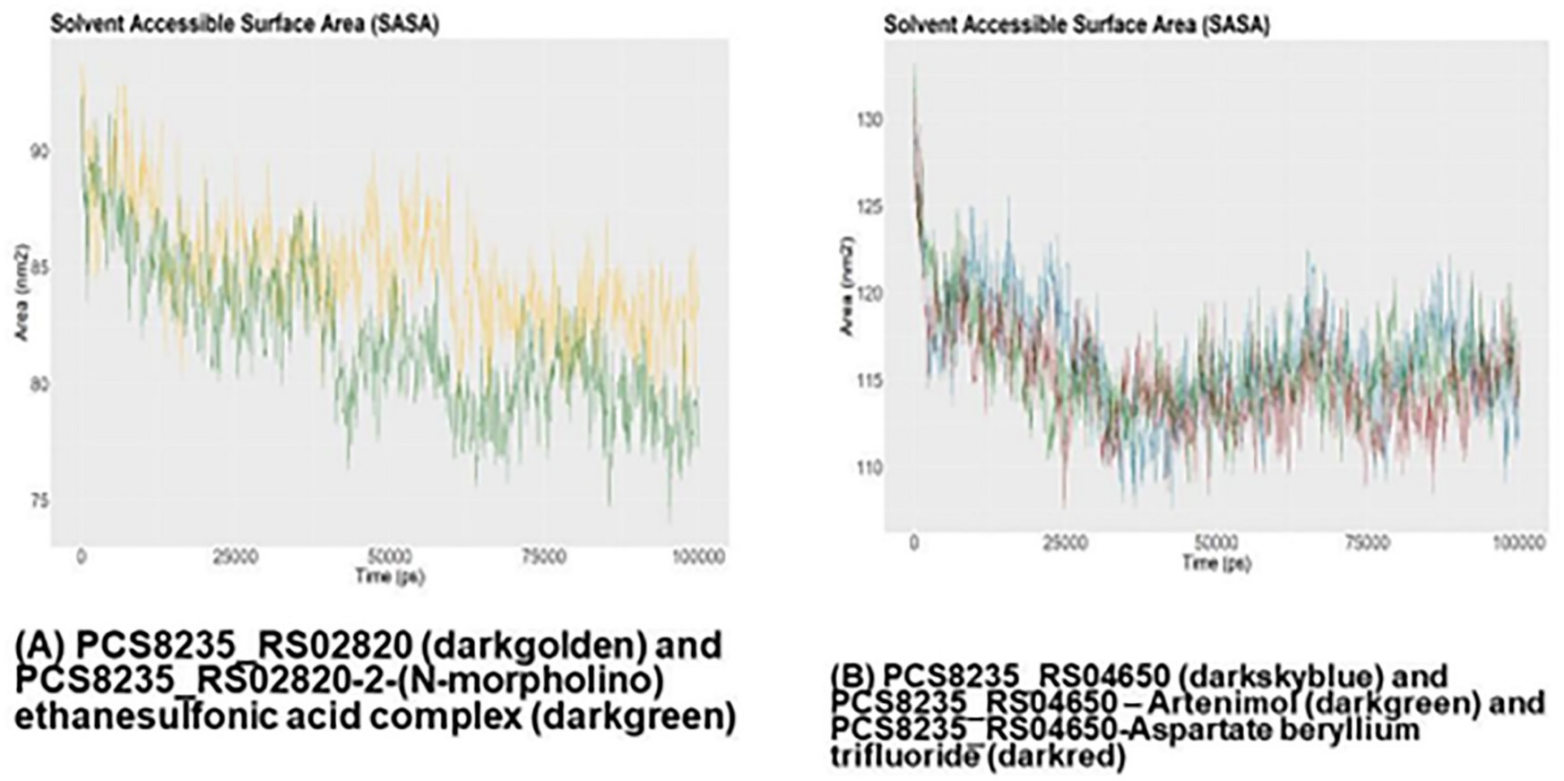
SASA calculation of (A) PCS8235_RS02820 (golden) and PCS8235_RS02820-2-(N-morpholino) ethanesulfonic acid complex (green) (B) PCS8235_RS04650 (blue) and PCS8235_RS04650 –Artenimol (green) and PCS8235_RS04650-Aspartate beryllium trifluoride (red) at 100ns.

**Fig 10:**
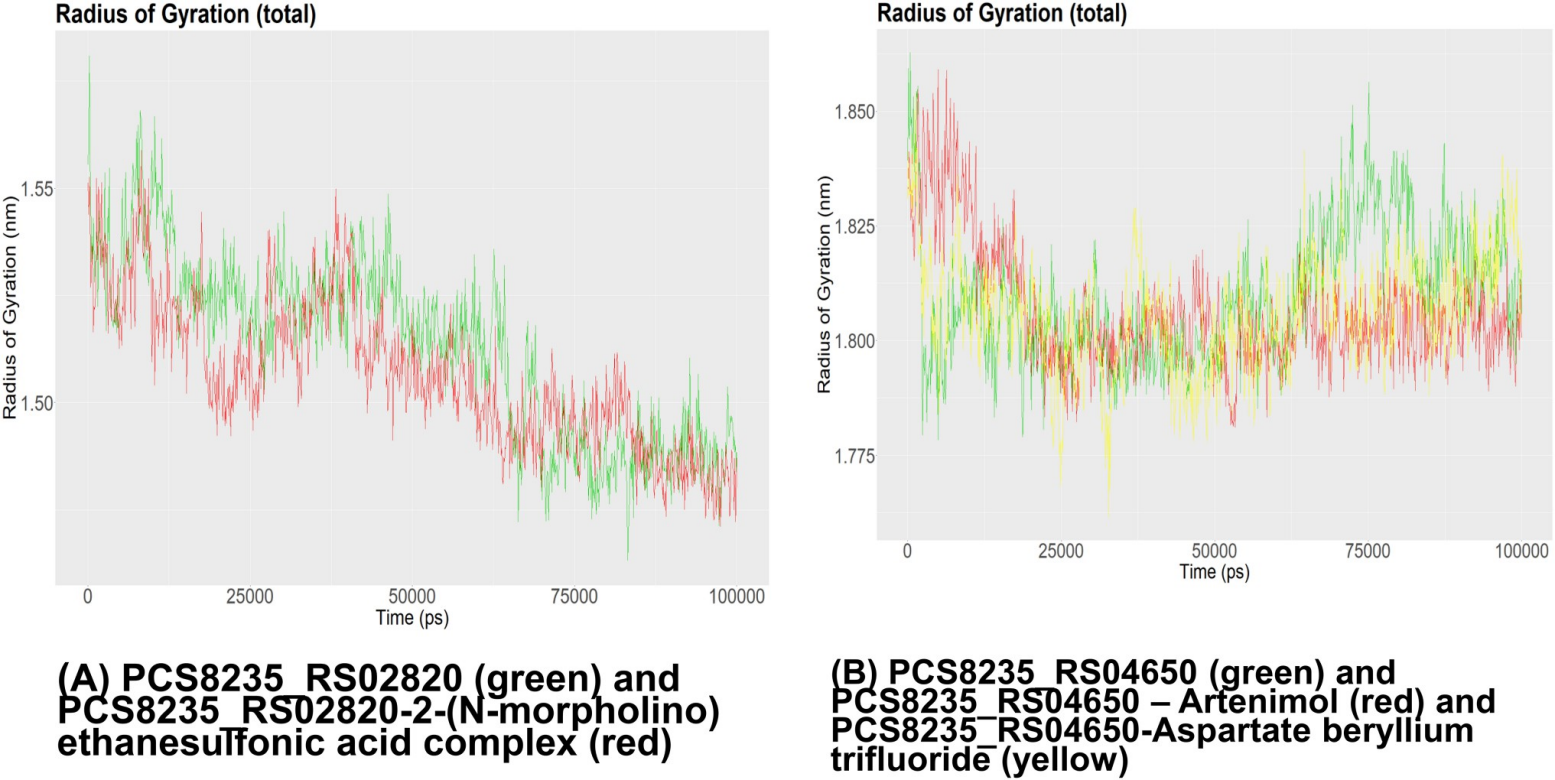
Rg measurement of (A) PCS8235_RS02820 (green) and PCS8235_RS02820-2-(N-morpholino) ethanesulfonic acid complex (red) (B) PCS8235_RS04650 (green) and PCS8235_RS04650 –Artenimol (red) and PCS8235_RS04650-Aspartate beryllium trifluoride (yellow) at 100 ns.

The RMSD graph of PCS8235_RS02820 and PCS8235_RS02820-drug complex demonstrated that the backbone of the proteins started to deviate around 12 ns and overlapped around 75 ns (Fig 7). The RMSF and SASA graphs of this protein showed that the binding of the drug altered mobility and solvent accessibility in the protein (Figs 8 and 9). Rg analysis unveiled that the compactness of the protein and drug-protein complex was most of the time similar during the simulations (Fig 10). For PCS8235_RS04650 and PCS8235_RS04650-drug bound complexes also gave similar results. The RMSD backbones did not overlap most of the time through 100ns. The RMSF values or mobility with protein compactness (Rg) of the structures altered due to drugs. Slight solvent accessibility was observed in SASA analysis.

## Discussion

*S*. *pneumoniae* has been the prime cause of death via community-acquired pneumonia (CAP) worldwide [1]. Combating the mortality and morbidity rate of CAP is becoming more challenging due to the increasing prevalence of antimicrobial resistance [57]. Following the drastic rise in antimicrobial resistance, current research on producing new antibiotics seems time-consuming, and costly. For several years, genomics and evolutionary studies on this organism were endeavored to determine new druggable targets [58, 59]. Yet, few studies have been published on the unexplored realm of proteins known as HPs. About nearly 30% of *S*. *pneumoniae* encodes HPs. Some homologs of HPs are conserved within various streptococci species [60]. In the present study, while analyzing the core genome of the *Streptococcus* species, we have found 498 core genes among 22 Streptococci species where 28 belonged to HPs (around 5.6%). Since they are widely distributed in the *Streptococcus* genus through evolution, they might be critical for the survival of the bacteria. Proper characterization of these HPs could give plausible targets against *S*. *pneumoniae* pathogen. Previous studies demonstrated that HPs play a vital role in biofilm formation, pathogenesis, and virulence of tooth decaying *S*. *mutans* [61] whereas, in this study, we have also identified the protein PCS8235_RS05215 that plays a key role in biofilm formation. Understanding the functional annotation of these HPs is essential to comprehending the molecular and biological mechanisms of all species. Bioinformatics approaches aiding functional annotation and computational drug designing can be a better way to identify the potential drug targets from these HPs.

When the core HPs went under gene ontology-based characterization, 75% of the proteins revealed their characteristic. Most of them were enzymes and binding proteins. Enzymes are involved in several biochemical reactions and self-defense mechanisms playing a key role in bacterial survival inside the host. Here, protein PCS8235_RS3915 and PCS8235_RS4650 were involved in hydrolase activity. Protein PCS8235_RS3915 is a DNA repair RadC homolog while PCS8235_RS4650 is a haloacid dehalogenase homolog. Haloacid dehalogenase homologs are a superfamily of enzymes involved in cellular functions starting from amino acid biosynthesis to detoxification [62, 63]. In prokaryotes, DNA repair RadC homolog helps in DNA damage repair after UV and X-ray radiation [64]. In the same way, protein PCS8235_RS2800 is related to branched-chain amino acid aminotransferase activity and protein PCS8235_RS6025 is associated with methyltransferase activity. Hence, enzymes can be assumed as a druggable target for different therapeutics. The preceding proteins (PCS8235_RS01335, PCS8235_RS01340, PCS8235_RS02405, PCS8235_RS03345, PCS8235_RS06025 and PCS8235_RS06945) also take part in nucleic acid-binding. Other proteins (PCS8235_RS02405, PCS8235_RS03345, PCS8235_RS06025, and PCS8235_RS06945) were somehow involved in the central dogma of the bacterial pathogen. Protein PCS8235_RS05215 denoted transcriptional regulator YlbF, YmcA that formed a complex with YaaT regulated competence and biofilm formation in Bacillus subtilis [65]. Furthermore, targeted proteins must be essential to the concerned pathogen since essential genes strongly support the cellular life of living microorganisms [66]. For the study, more than half of the proteins were identified as essential proteins of *S*. *pneumoniae*. Later, the virulence and pathogenicity of the essential HPs were noted. Out of the 19 proteins analyzed for virulence and pathogenic characteristics, 21% were found to be both virulent and pathogenic.

Moreover, the integrated roles of the bacterial proteins were elucidated via protein-protein interactions. Protein-protein interaction is required to understand the specific functions of bacterial protein which might be influenced by the neighboring proteins. This helps in finding its significance in bacterial physiology. In the provided network, the protein PCS8235_RS05845 formed the highest nodes (8 interactions) with other HPs that might have critical cellular functions and the potential to go under unconventional protein secretions. Most of the HPs play a physiological role in the cytoplasmic region and have hydrophilic properties. Therefore, they can be considered as drug targets rather than vaccines [29, 67]. This cytoplasmic characteristic was evaluated by subcellular localization and physicochemical properties. PCS8235_RS05845 also has TRP structural motif along with virulent and pathogenic properties. Previous studies reported that some TRP-containing proteins are involved directly in virulence-associated functions, especially, in maintaining proper functions of the type III secretion system and class II chaperones [67]. The next highest interactive protein is PCS8235_RS02595 (7 interactions). Although this protein is uncharacterized still now, its interaction with other proteins can suggest its significant role in the network. PCS8235_RS02595 is interconnected to Pneumococcal vaccine antigen A (PCS8235_RS03660), cell division protein Zap A (PCS8235_RS00650), putative Anti-CRISPR (PCS8235_RS04815) and Regulator of chromosomal segregation (RocS) (PCS8235_RS03470) proteins. Zap A is a Z-ring-associated protein found in Bacillus subtilis [68]. This protein binds to tubulin-like protein FtsZ at cytokinesis and therefore takes part during bacterial cell division [69]. Putative Anti-CRISPR may inhibit the bacterial CRISPR-Cas system. RocS participates in chromosome segregation and nucleoid protection in *S*. *pneumoniae* [70].

The druggability of the essential HPs was checked to ensure the proteins’ susceptibility toward the active site of the small inhibitor molecules. In other studies, the drug targets in *S*. *pneumoniae* were explored; however, it has only screened the potential drug targets for the pathogen [71]. Here, only the compounds with both druggability and essentiality are chosen for further analysis. The 3D structure of the targeted proteins was then analyzed and the best model was selected for molecular docking. The basis of docking was to identify the relationship between the hypothetical proteins and available druggable ligands. A blind docking test for each of the whole proteins with expected drug candidates reveals satisfactory drug affinity towards the ligands. The strongest binding affinity could be noticed between PCS8235_RS04650 and Artenimol with the binding affinity of -9.5 kcal/mol. MD simulations also support that these druggable compounds will change the targeted bacterial proteins by impeding their general physiology. Therefore, this predictive essential protein could be an eligible drug target for the development of new pneumococcal therapeutics.

## Conclusion

This study focused on the HPs that are found in the core genome of *S*. *pneumoniae* and have a major role in the survival, virulence, and pathogenesis of the pathogen. The majority of the observed proteins are mainly involved in bacterial transcription and translation while a high proportion of the proteins were recognized as essential proteins. Hence they make up good targets for broad spectrum anti-streptococcal drug development.

## Supporting information

S1 Data List of all 498 genes that constitute the core genome of *Streptococcus sp*. (XLSX)

S2 Data The core proteome of *Streptococcus* strains. (XLSX)

S3 Data Result of gene ontology analysis (biological processes, molecular functions, and cellular components) of the 28 hypothetical proteins. (XLSX)

S4 Data Result of virulence and pathogenicity analysis. (XLSX)

S5 Data Functional enrichment of the interconnected proteins generated by STRING webserver. (DOCX)

S6 Data Ramachandran plot statistics of the hypothetical proteins PCS8235_RS02820 and PCS8235_RS04650. (XLSX)
