# Supplementary material for: Exploration of Streptococcus core genome to reveal druggable targets and novel therapeutics against S. pneumoniae
Source: PLoS One. 2022 Aug 18;17(8):e0272945. doi: 10.1371/journal.pone.0272945 (PMC9387852; doi:10.1371/journal.pone.0272945)

Supplementary Table 5 Functional enrichment of the interconnected proteins generated by STRING


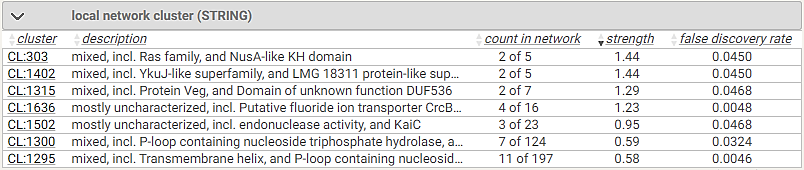

Supplement: S5 Data — (DOCX) [file pone.0272945.s005.docx]
